# N-Acetylcysteine Attenuates Hexavalent Chromium-Induced Hypersensitivity through Inhibition of Cell Death, ROS-Related Signaling and Cytokine Expression

**DOI:** 10.1371/journal.pone.0108317

**Published:** 2014-09-23

**Authors:** Yu-Hsuan Lee, Shih-Bin Su, Chien-Cheng Huang, Hamm-Ming Sheu, Jui-Chen Tsai, Chia-Ho Lin, Ying-Jan Wang, Bour-Jr Wang

**Affiliations:** 1 Department of Environmental and Occupational Health, National Cheng Kung University Medical College, Tainan, Taiwan; 2 Department of Occupational Medicine, Chi-Mei Medical Center, Tainan, Taiwan; 3 Department of Leisure, Recreation and Tourism Management, Southern Taiwan University of Science and Technology, Tainan, Taiwan; 4 Department of Medical Research, Chi Mei Medical Center, Liouying, Tainan, Taiwan; 5 Department of Emergency Medicine, Chi-Mei Medical Center, Tainan, Taiwan; 6 Department of Child Care and Education, Southern Taiwan University of Science and Technology, Tainan, Taiwan; 7 Department of Dermatology, National Cheng Kung University Medical College, Tainan, Taiwan; 8 Institute of Clinical Pharmacy, National Cheng Kung University, Tainan, Taiwan; 9 Division of Urology, Department of Surgery, Chi Mei Medical Center, Liouying, Taiwan; 10 Department of Biotechnology, Southern Taiwan University of Science and Technology, Tainan, Taiwan; 11 Department of Biomedical Informatics, Asia University, Taichung, Taiwan; 12 Department of Cosmetic Science and Institute of Cosmetic Science, Chia Nan University of Pharmacy and Science, Tainan, Taiwan; Taipei Medicine University, Taiwan

## Abstract

Chromium hypersensitivity (chromium-induced allergic contact dermatitis) is an important issue in occupational skin disease. Hexavalent chromium (Cr (VI)) can activate the Akt, Nuclear factor κB (NF-κB), and Mitogen-activated protein kinase (MAPK) pathways and induce cell death, via the effects of reactive oxygen species (ROS). Recently, cell death stimuli have been proposed to regulate the release of inflammatory cytokines, such as tumor necrosis factor-α (TNF-α) and interleukin-1 (IL-1). However, the exact effects of ROS on the signaling molecules and cytotoxicity involved in Cr(VI)-induced hypersensitivity have not yet been fully demonstrated. N-acetylcysteine (NAC) could increase glutathione levels in the skin and act as an antioxidant. In this study, we investigated the effects of NAC on attenuating the Cr(VI)-triggered ROS signaling in both normal keratinocyte cells (HaCaT cells) and a guinea pig (GP) model. The results showed the induction of apoptosis, autophagy and ROS were observed after different concentrations of Cr(VI) treatment. HaCaT cells pretreated with NAC exhibited a decrease in apoptosis and autophagy, which could affect cell viability. In addition, Cr (VI) activated the Akt, NF-κB and MAPK pathways thereby increasing IL-1α and TNF-α production. However, all of these stimulation phenomena could be inhibited by NAC in both of *in* *vitro* and *in* *vivo* studies. These novel findings indicate that NAC may prevent the development of chromium hypersensitivity by inhibiting of ROS-induced cell death and cytokine expression.

## Introduction

Chromium is ubiquitous in the environment and can be found in pigments, chrome-plated metals, tanned shoe leather, cement, detergents, and industrial chromium waste dumps [1]. Chromium has several oxidation states, including Cr(II), Cr(III), Cr(IV), Cr(V) and Cr(VI), but only Cr(III) and hexavalent chromium (Cr(VI)) are stable. In general, Cr(III) diffuses through the skin at a much lower rate than Cr(VI), which may account for its lower dermatological toxicity. However, once Cr(VI) penetrates the skin, it is reduced to Cr(III) [2], [3]. The trivalent form binds to keratinocytes and immune cells of the skin, and this is most likely form that is ultimately responsible for dermal toxicity [1]. The intracellular reduction of Cr(VI) is associated with the production of reactive oxygen species (ROS). ROS has been implicated as the cause of many human disorders and in the toxicity of numerous xenobiotics [4]. In the skin, ROS play an important role in the pathogenesis of allergic contact dermatitis (ACD) [5], [6]. Metallic allergens such as nickel and chromium are both producers of ROS and have been proved to induce ACD [7], [8]. Through redox cycling reactions, chromium, cobalt and other metals produce reactive radicals to result in toxic effects but this is not true for lead. Lead is a redox inactive metal and it isn’t the common agent to induce ACD [9], [10]. Following dermal exposure, chromium causes two types of dermatological toxicity. The most widely known reaction is sensitization and the elicitation of ACD. Chromium hypersensitivity is common in both the general population and certain occupation-related workers, with prevalences of approximately 0.5% and 4–5% in European populations and cement workers, respectively [11], [12]. In fact, chromium hypersensitivity is an important occupational skin disease among cement workers.

Exposure to chemical agents can result in cell damage and death. The survival or death of the exposed cells is often determined by their proliferative status and ability to induce proteins that either promote or inhibit cell death processes [13]. Different modalities of cell death (apoptosis, necrosis, autophagy) contribute to the pathophysiology of different human disorders [14]. In general, apoptosis is an active process of cell destruction with specific defining morphologic and molecular features that leads to orderly cell disassembly. ROS can cause cellular apoptosis via both the mitochondria-dependent and mitochondria-independent pathways [15]. In contrast, autophagy is a protein degradation system in which cellular proteins and organelles are sequestered, delivered to lysosomes, and digested by lysosomal hydrolases. In normal cells, autophagy functions maintain homeostasis by eliminating excessive or unnecessary proteins [16]. In recent years, the role of autophagy as an alternative cell death mechanism has been a topic of debate. A complex of signaling pathways control the induction of autophagy in different cellular contexts. ROS were recently shown to activate starvation-induced autophagy, antibacterial autophagy, and autophagic cell death [17], [18].

Apoptotic cell death has been suggested to play a key role in numerous skin inflammatory diseases. In this regard, studies in mouse models have emphasized the role of increased keratinocyte apoptosis in cutaneous inflammation [19]. In addition, there is a direct link among autophagy, cell death, antigen processing, and the generation of inflammatory and immune responses [20]. During these processes, ROS-regulated redox-sensitive protein kinases and transcription factors (for example Nuclear factor κB (NF-κB), Mitogen-activated protein kinase (MAPK) and Akt pathway) may affect the release of cytokines, such as tumor necrosis factor (TNF-α) and interleukin-1(IL-1) [21]–[24]. The release of these cytokines is suggested to be a central and early event in the progression of ACD [25], [26]. When ROS are generated, antioxidants, including enzymatic antioxidant systems and non-enzymatic antioxidant systems [27], [28], counteract the oxidative effects of these ROS to protect the body. N-acetylcysteine (NAC), a thiol and mucolytic agent, is an effective precursor of cysteine that has been used for research on the role of ROS in many disease processes [29].

In our previous studies, we found that Cr(VI) could increase ROS formation, activate the Akt, NF-kB, and MAPK pathways and increase the production of cytokines, including TNF-α and IL-1α. The release of these cytokines from keratinocytes is considered a key element of the pathogenesis of contact hypersensitivity [30]. In addition, we further revealed that NAC could inhibit chromium hypersensitivity in a coadjuvant chromium-sensitized albino guinea pig model by counteracting the formation of ROS [3]. However, the exact effects of ROS on the signaling molecules and cytotoxicity involved in Cr(VI)-induced hypersensitivity have not been extensively studied. Currently, we have extended our analysis to study the effects of NAC on attenuating the Cr(VI)-triggered ROS signaling in both normal keratinocyte cells and guinea pig model. Our primary goal is to understand the involvement of apoptosis and autophagy by which ROS regulates the signaling pathways involved in Cr(VI)-induced hypersensitivity.

## Materials and Methods

### Chemicals and antibodies

Dulbecco’s modified Eagle’s medium (DMEM), penicillin, and streptomycin were purchased from Cibco BRL (Paisley, Scotland, U.K.). Dimethyl sulphoxide (DMSO) and EDTA were purchased from Sigma Chemical (Poole, Dorset, U.K.). Potassium dichromate (K_2_Cr_2_O_7_) and NAC were obtained from Merck Chemical Co. (Darmstadt, Germany). MTT (3-(4,5-dimethylthiazol-2-yl)-2,5-diphenyltetrazolium bromide) was purchased from Sigma-Aldrich, Inc. (St. Louis, MO, U.S.A.). The antibodies for detecting anti-poly-(ADP-ribose) polymerase (PARP) antibody were obtained from Millipore (Billerica, MA, U.S.A.); anti-caspase-3 and anti-cleaved-caspase-3 antibodies were obtained from Epitomics (Burlingame, CA, U.S.A.); anti-LC3 antibody was obtained from Abgent (San Diego, CA, U.S.A.). Akt, phospho-Akt, p65, phospho-p65, IκB-α, ERK1/2, phospho-ERK1/2, p-38, phospho-p38, JNK and phospho-JNK were purchased from Cell Signaling (Beverly, MA, U.S.A.); GAPDH was obtained from Abcam Inc.(Cambridge, MA, U.S.A.); phospho- IkB-α was purchased from Upstate Biotechnology (Lake Placid, NY, U.S.A.). Horseradish peroxidase (HRP)-conjugated anti-mouse and anti-rabbit secondary antibodies were purchased from Jackson ImmunoResearch Laboratories Inc. (West Grove, PA, U.S.A.). TNF-α and IL-1α antibodies were purchased from Santa Cruz Biotechnology (Santa Cruz, CA, U.S.A.).

### HaCaT cells culture

HaCaT cells were a gift from Professor H.M. Sheu (Department of Dermatology, National Cheng Kung University, Taiwan) and were cultured according to previous methods [31]. In brief, the HaCaT cells were cultured in Dulbecco’s Modified Eagle Medium (DMEM) (GIBCO, Grand Island, NY) supplemented with antibiotics, including 100 IU/ml penicillin and 1000 µg/ml streptomycin (Life Technology, Grand Island, NY), and 10% heat-inactivated fetal calf serum (HyClone, South Logan, Utah). The cells were incubated at 37°C in a humidified atmosphere containing 5% CO_2_. Exponentially growing cells were detached by 0.1% trypsin-EDTA (Gibco) in PBS. For the treatment of NAC, 1 M stock solution was added to the culture medium in a concentrated form, gently mixed for 1 hr and treated with different concentrations of Cr(VI). The cultures were then incubated for different periods of time, as indicated in the figures.

### MTT cell viability assay

Using previously described methods [32], 5×10^4^ HaCaT cells were placed in each of the 96 wells and treated with NAC and different concentrations of Cr(VI) for 24 hrs. Then, 100 µl of 0.5 mg/ml MTT was added and, finally, the solution was incubated at 37°C for 4 hrs. After incubation, the MTT was removed, and DMSO was added to dissolve the formazan. The optical density was measured by an ELISA reader (Emax, Molecular Devices, Sunnyvale, CA, U.S.A.) at 570 nm and the amount of formazan generated was calculated.

### Detection of early apoptosis using Annexin V staining

Apoptosis was assessed by observing the translocation of phosphatidyl serine to the cell surface, detected with an Annexin V apoptosis detection kit (Calbiochem, San Diego, CA, U.S.A.) as described previously [33]. Cells were pretreated with NAC (20 mM) and/or exposed to Cr(VI) in a concentration manner. After 24 hrs of exposure, cells were trypsinized, washed with 1× PBS and centrifuged at 3000 rpm for 5 min. Cells were resuspended in 100 µl of 1× Annexin V-binding buffer (10 mM HEPES (pH 7.4), 0.14 M NaCl and 2.5 mM CaCl_2_) that contained 5 µl of Annexin V-FITC (Becton Dickinson, San Jose, CA, U.S.A.) and were incubated at room temperature for 15 min. The 1× binding buffer (400 µl) was added to stop the reaction, and the stained cells were collected for flow cytometry analyses.

### Detection and quantification of acidic vesicular organelles with acridine orange staining

Cell staining with acridine orange (Sigma Chemical Co. Poole, Dorset, U.K.) was performed according to the published procedures [34], [35]. A final concentration of 1 µg/ml was added for a period of 20 min. Flow cytometric analysis was used to detect the acidic vesicular organelles (AVOs), which are a characteristic of autophagy [34].

### Reactive oxygen species detection by fluorescence measurement

ROS production was monitored by a fluorescence microplate reader using the Amplex Red reagent (Molecular Probes/Invitrogen, Carlsbad, CA, U.S.A.). The principle of this test is that hydrogen peroxide (H_2_O_2_) will react with Amplex Red (10-acetyl-3,7-dihydroxyphenoxazine) in the presence of peroxidase to produce its red-fluorescent oxidation product, resorufin. Resorufin could be detected fluorometrically. Experimentally, HaCaT cells (5×10^4^/well) were seeded in 96 multiwell plates. Cells were pretreated with NAC at a final concentration of 50 mM for 1 hr. After washing with PBS, cells were exposed to 15 µM Cr(VI) and incubated with Amplex Red reagent in a reaction buffer containing horseradish peroxidase. Fluorescence was recorded in a microplate reader (Thermo, Fisher Scientific, Waltham, MA, U.S.A.) set to 530 nm excitation and 590 nm emission wavelengths. A fluorescence microplate reader was used to take a time zero (Ft0) point. At each time point, the fluorescence intensity was again determined. For the data analysis, the percentage increase in fluorescence per well was calculated by the formula [(Ftx-Ft0)/Ft*100], where Ftx is the fluorescence at time x minutes and Ft0 is fluorescence at time 0 minute [36]. The fluorescence data reflected the subtraction of untreated control cell values and is presented as the mean ± SD of sextuplicate samples.

### Western blot analysis

Total cellular protein lysates were prepared by harvesting cells in a protein extraction buffer (10 mM Tris-HCl pH 7, 140 mM sodium chloride, 3 mM magnesiumchloride, 0.5% [w/v] NP-40, 2 mM phenylmethylsulfonyl fluoride, 1% [w/v] aprotinin and 5 mM dithiothreitol) for 1 hr at 4°C [37], [38]. After treatment, proteins isolated from the HaCaT cells were loaded at 50 µg/lane on 12% (w/v) sodium dodecylsulfate-polyacrylamide gels, subjected to electrophoresis, blotted, probed using antibodies and detected using a chemiluminescence (ECL) detection system (Millipore, WBKLS0500). In the following experiments, proteins were used for the determination of apoptosis, autophagy, and the Akt, NF-κB and MAPK pathways. GAPDH expression was used as the protein loading control. The densities of the bands were quantified with a computer densitometer (AlphaImager 2200 System Alpha Innotech Corporation, San Leandro, CA, USA).

### mRNA expression of TNF-α and IL-1α

HaCaT cells were exposed to NAC (20 mM) for 1 hr before treatment with a range of Cr(VI) concentrations (15, 30, and 60 µM). After 4 hrs of exposure, the total RNA was extracted using a TRIZOL reagent (Invitrogen, Carlsbad, CA, U.S.A.) according to the supplier’s protocol. RNA concentrations and 260/280 ratios were measured by a spectrometer (Beckman, DU640B, San Diego, CA, U.S.A.). Reverse transcription (RT) was performed using Superscript II (Life Technologies Inc., Carlsbad, CA, U.S.A.). Oligonucleotide primers that correspond to human TNF-α, IL-1α and β-actin were purchased from Stratagene (La Jolla, CA, U.S.A.). After the creation of cDNA, polymerase chain reaction (PCR) was used to amplify the corresponding amount of cDNA. For analysis, 10 µl of each PCR product was separated by electrophoresis in a 1.5% agarose gel and then stained with ethidium bromide. The gel was then photographed under ultraviolet light, and the densities of the bands were quantified with AlphaImage 2200 System. β-actin mRNA was used as a loading control. Primers for TNF-α, IL-1α and β-actin were as follows: TNF-α, forward 5′-AGCCCACGTCGTAGCAAACCACCAA- 3′ and reverse 5′-ACACCCATTCCCTTCACAGAGCAAT-3′; IL-1α, forward 5′-GGAAGGTTCTGAAGAAGAGACG-3′ and reverse 5′-GAGGTTGGTCTCACTACCTGTGAT- 3′; and β-actin, forward 5′-AAGAGAGGCATCCTCACCCT-3′ and reverse 5′-TACATGGCTGGGGTGTTGAA-3′.

### ELISA for IL-1α detection

HaCaT cells (6×10^5^) were incubated with DMEM for 24 hrs in 6-well plates, then exposed to NAC (20 mM) for 1 hr before treatment with a range of Cr(VI) concentrations (15, 30, and 60 µM) and incubated for an additional 24 hrs in serum- free DMEM. The supernatant was collected for IL-1α detection. IL-1α levels in the HaCaT cells supernatants were measured by enzyme-linked immunosorbant assay (ELISA) (Quantikine, R&D systems, Minneapolis, MN, USA.) according to the manufacturer’s instructions. The optical density of the peroxidase substrate (tetramethylbenzidine) was read using an ELISA reader (Emax, Molecular Devices, Sunnyvale, CA, U.S.A.) at 450 nm. Based on the standard curve, the concentrations of IL-1α in each sample were determined.

### Administration of NAC and treatment of Cr(VI) injected albino GP

Female albino Hartley strain guinea pigs (GP) from the Animal Center of the National Taiwan University were used in this study. The animals were housed in the animal center of the National Cheng-Kung medical center in standard polycarbonate cages with free access to water and pellet food. Three groups of GP (A, B, and C; n = 3 per group) were used. Group A and Group B were fed only ordinary food, while Group C was fed ordinary food and 1200 mg/kg/day of NAC. Each GP was weighed daily, and the daily NAC dosage was calculated and administered. After a period of 2 weeks, Group A was injected with saline only, while Group B and C were injected with 0.3 ml of saline solution containing 2 mg/ml Cr(VI), using the coadjuvant chromium sensitization method, described by van Hoogstraten et al. [39]. Briefly, an anesthetized GP received 0.2 ml of saline solution into the shaved sites of the dorsal skin on both thighs (0.1 ml/thigh) and 0.05 ml into the pinna of each ear by intradermal injections. Forty-eight hours after the injection, the dorsal skin (1.5×1.5 cm) on each thigh from each GP was excised to determine the protein expression of the Akt, NF-kB and MAPK pathways. Following the method of a previous study [3], the excised dorsal skin was prepared for protein isolation and Western blot analysis.

### Ethics statement

All experiments on albino GP were performed according to the guidelines of our institute (Guide for Care and Use of Laboratory Animals, National Cheng Kung University Medical College). The animal use protocol listed below has been reviewed and approved by the Institutional Animal Care and Use Committee of National Cheng Kung University, Taiwan (Approval No: 100127). All procedures were performed under anesthesia and all efforts were made to minimize suffering and the number of animals used. For the GP anesthesia, a 4∶3 mixture of ketamine (Pfizer, Inc., NY, U.S.A.) and Rompun (Bayer Pharma AG, Leverkusen, Germany) was injected intramuscularly at 1 µl/g body weight.

### Immunohistochemical (IHC) staining analysis

Paraffin-embedded tissue sections (5 µm) were dried, deparaffinized, and rehydrated. The hydrated tissue sections were steamed in Dako target retrieval buffer (pH 9.0) (DAKO Corp, Carpinteria, CA, U.S.A.) for 30 min for antigen retrieval, treated with 3% hydrogen peroxide, and blocked with a DAKO antibody diluent (DAKO Corp, Carpinteria, CA, U.S.A.) for 1 h at room temperature. The tissue sections were incubated with aliquots of antibodies (Santa Cruz Biotechnology, Santa Cruz, CA, U.S.A.) against TNF-α (1∶500) and IL-1α (1∶100) at 4°C overnight. After washing, aliquots of biotinylated secondary antibodies (Biotinylated Link Universal) were added. Color development was performed using a labeled streptavidin biotin plus horseradish peroxidase kit (DAKO Corp., Carpinteria, CA, U.S.A.) Finally, the slides were counterstained using hematoxylin and examined under light microscopy.

### Statistical analysis

All data represented the mean ± SD of at least three independent culture experiments. Experimental data were analyzed using Student’s t test. Differences were considered statistically significant when the *p* value was less than 0.05. Images are representative of three or more experiments.

## Results

### Protective effect of NAC on cell viability

**Figure 1:**
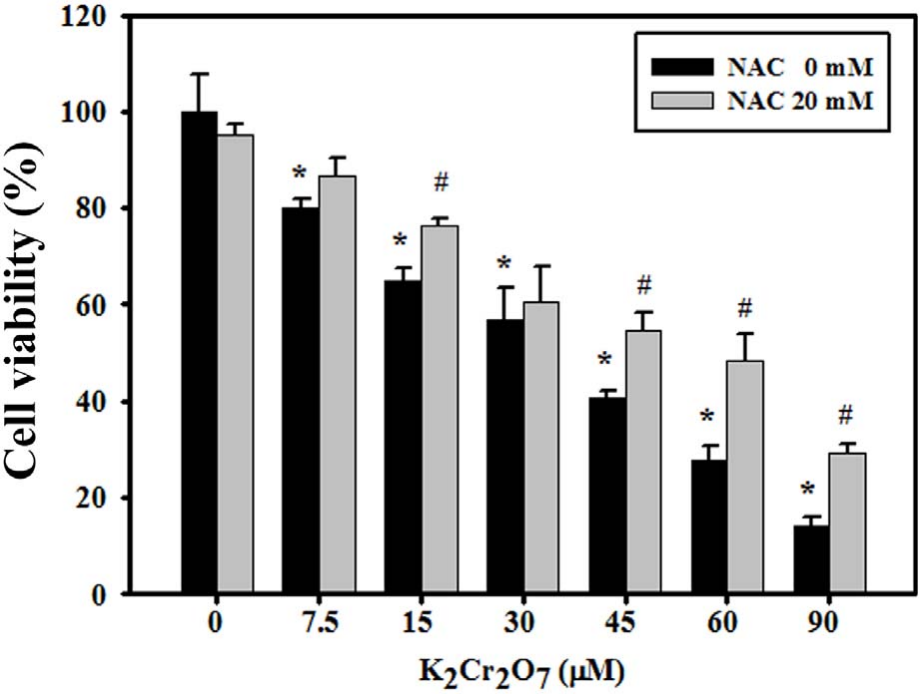
Effects of NAC on Cr(VI) induced cytotoxicity. Cells were pretreated with NAC (20 mM), cultured for 1 hr and incubated in the presence of varying concentrations of Cr(VI) for 24 hrs. Cell viability was determined by MTT assays. The data are the mean ± SD values of triplicate measurements from a representative of three independent experiments. (*Significant versus Control group; *p*<0.05; #Significant versus Cr(VI)-treated Group; *p*<0.05).

The viability of cultured cells was determined utilizing an MTT, assay which was based on the conversion of MTT to a formazan by intracellular dehydrogenases [40]. The results showed a dose-dependent effect. When the concentration of Cr(VI) increased, the viability of HaCaT cells decreased. The IC_50_ value was between 30∼45 µM. One hour after NAC pretreatment, the viability of cultured cells was significantly higher than when treated with the same Cr(VI) dosage alone (Fig. 1).

### NAC attenuated Cr(VI)-induced apoptotic cell death

**Figure 2:**
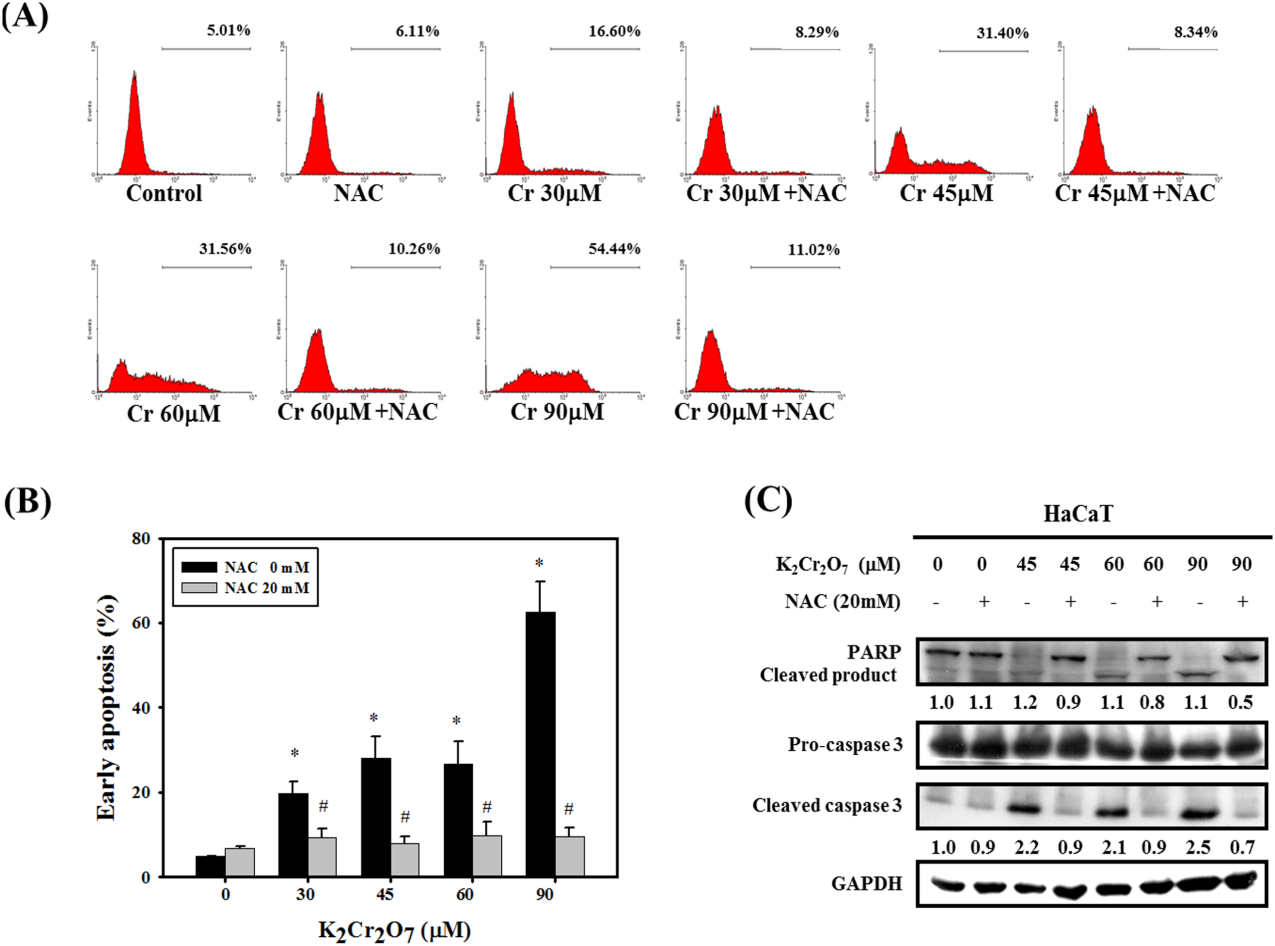
NAC attenuated Cr(VI)-induced apoptotic cell death. (A) Flow cytometry analysis indicating the rate of apoptosis in HaCaT cells treated with Cr(VI) or Cr(VI) plus NAC. Early apoptosis were detected by an Annexin V apoptosis detection kit. (B) Quantification of early apoptosis in HaCaT cells. The results are presented as the mean ± SD in duplicate in three independent experiments. (*Significant versus Control group; *p*<0.05; #Significant versus Cr(VI) -treated Group; *p*<0.05). (C) Western blotting of PARP, cleaved-PARP, procaspase 3 and cleaved-caspase 3. The level of total GAPDH protein was used as the loading control. Cells were pretreated with NAC (20 mM) for 1 hr prior to treatment with Cr(VI) for 24 hrs. Data are presented from three independent experiments.

To investigate the effects of NAC on Cr(VI)-induced apoptotic cell death, the level of early apoptosis was analyzed by flow cytometry with the Annexin V apoptosis detection kit. As shown in Figs. 2A & 2B, treatment with Cr(VI) for 24 hrs significantly increased the rate of apoptotic cell death. When 20 mM NAC was added as a pretreatment before Cr(VI) exposure, Cr(VI)-induced apoptosis was attenuated. In addition, Cr(VI)-induced apoptotic cell death was further confirmed by the accumulation of active forms of PARP and caspase 3. Treatment with Cr(VI) caused the cleavage of PARP and caspase 3, and this cleavage was inhibited by NAC (Fig. 2C). These results demonstrated that NAC effectively blocked Cr(VI)-induced apoptotic cell death.

### Effect of NAC on Cr(VI)-induced autophagy in HaCaT cells

**Figure 3:**
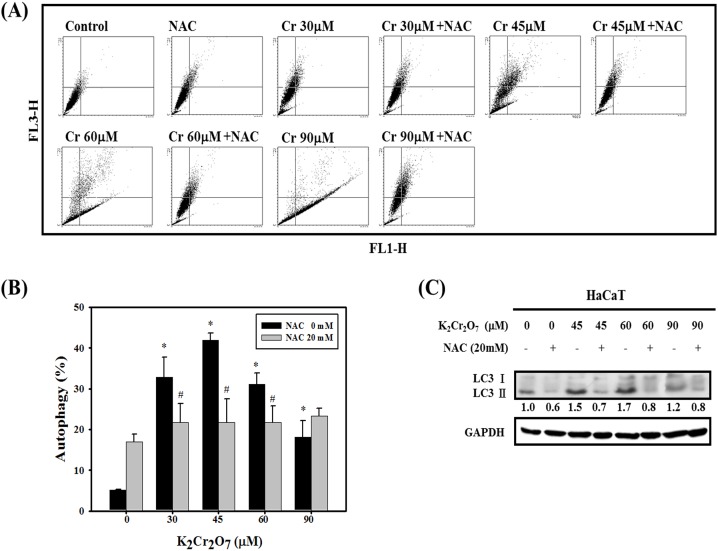
Effect of NAC on Cr(VI)-induced autophagy in HaCaT cells. (A) Development of AVOs in HaCaT cells. Detection of green and red fluorescence in AO-stained cells using flow cytometry. Cells were pretreated with NAC (20 mM), cultured for 1 hr and then incubated in the presence of different concentrations of Cr(VI) for 24 hrs. (B) Quantification of AVOs in HaCaT cells. Data are presented as the mean ± SD from three independent experiments. (*Significant versus Control group; *p*<0.05; #Significant versus Cr(VI) -treated Group; *p*<0.05). (C) The expression of autophagy proteins, LC3-I and LC3-II, and GAPDH were monitored after 24 hrs of Cr(VI) or Cr(VI) plus NAC exposure in HaCaT cells. Data are presented from three independent experiments.

Autophagy can regulate a number of cellular responses and be involved in the responses of cells towards various stresses, such as nutrient deprivation, oxidative stress and intracellular pathogens [41]–[43]. Cr(VI) exposure activates the autophagic process, as shown by the marked increase in acidic vesicular organelles (AVOs) (Figs. 3A, 3B). AVOs stain green/red with acridine orange and specify autophagy [44], [45]. However, pretreatment with NAC significantly reduced these increases in comparison to the Cr(VI) treatment group, except with the 90 µg/ml Cr(VI) exposure. Furthermore, we performed western blotting with lysates from HaCaT cells receiving different concentrations of Cr(VI) (Fig. 3C). Expression levels of the LC3-II protein, which is a marker of autophagy, increased with Cr(VI) treatment but was downregulated by NAC pretreatment. These findings suggested that Cr(VI) induced autophagy in HaCaT cells and this process was blocked by treatment with NAC.

### Cr(VI) treatment induced hydrogen peroxide/ROS generation in HaCaT cells, which were attenuated by NAC

**Figure 4:**
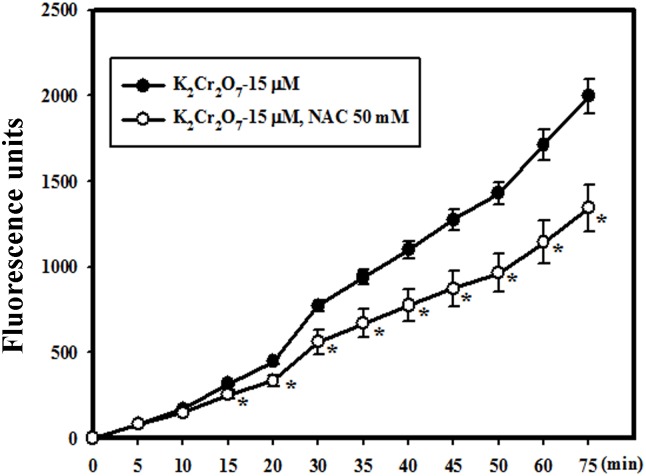
Cr(VI) treatment induced hydrogen peroxide/ROS generation in HaCaT cells which was inhibited by NAC. HaCaT cells were treated with 15 µM of Cr(VI) for various time periods (5, 10, 15, 20, 30, 40, 45, 50, 60, and 75 min). The amount of hydrogen peroxide produced in cells was determined using the oxidation of a fluorogenic indicator, Amplex Red (10-acetyl-3,7-dihydroxyphenoxazine). Pretreatment with NAC could suppress hydrogen peroxide/ROS generation. Fluorescence units reflected the subtraction of untreated cell values and were presented as the mean ± SD. (*Significant versus Cr(VI)-treated Group; *p*<0.05).

To evaluate whether cellular hydrogen peroxide production was involved in Cr(VI)-mediated cell signaling, we measured levels of ROS produced in HaCaT cells. First, we found that an exposure to Cr(VI) increased the hydrogen peroxide production. These findings correlate well with results showing Cr(VI) induced ROS generation in human lung cell types [46], [47]. In addition, pretreatment with NAC (50 mM) significantly reduced hydrogen peroxide production compared with the Cr(VI) treatment alone group (Fig. 4). This result is similar to the observations of Faurschou et al. and Young et al. [21], [36]. They treated HaCaT cells and primary human keratinocytes with TNF-α and showed that ROS formation increased, but this could be inhibited by NAC.

### Effect of NAC on Cr(VI)-stimulated activation of the Akt, NF-κB and MAPK pathways

**Figure 5:**
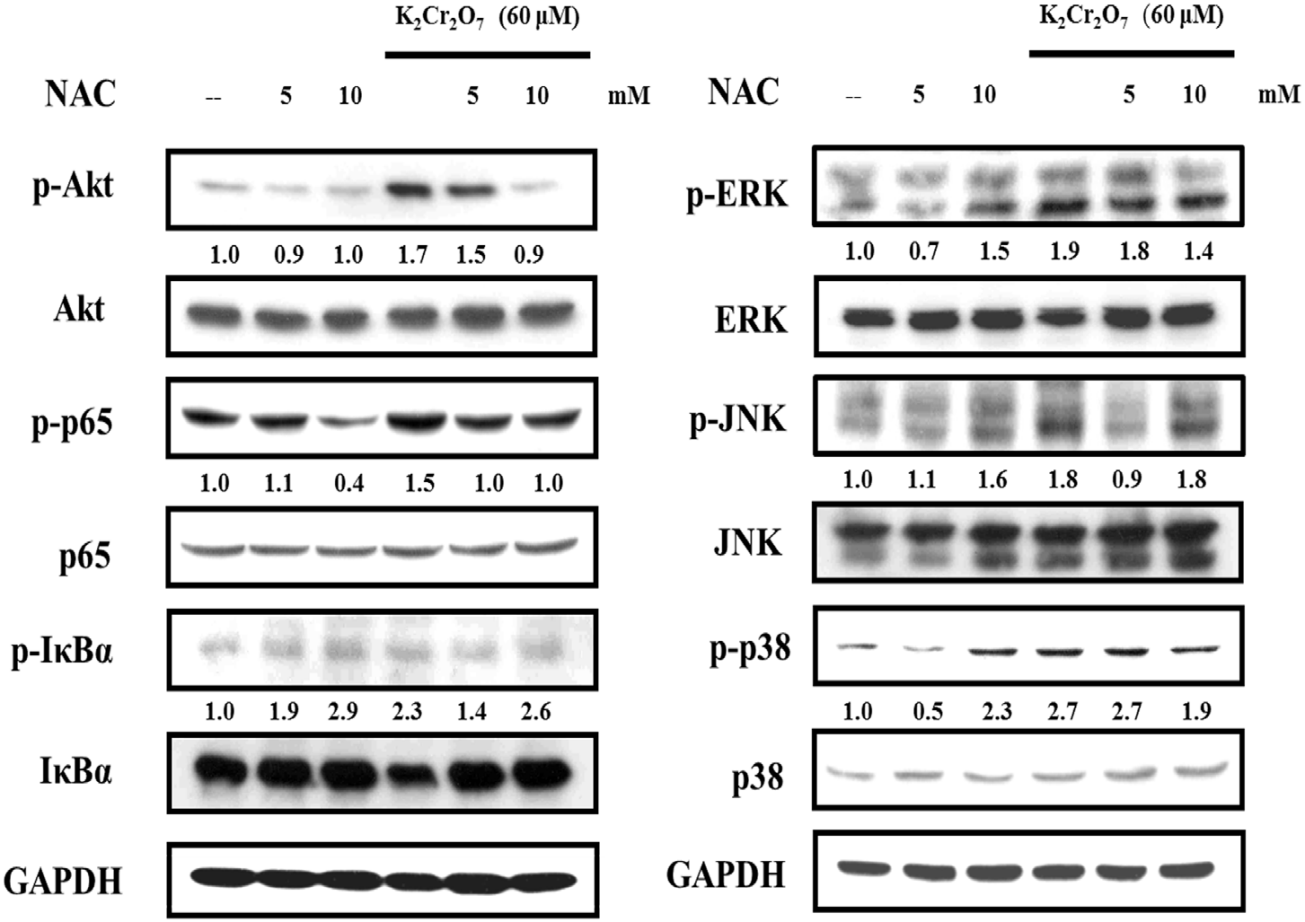
Akt-, NFκB-, and MAPK- related protein expression were analyzed by western blot. In three independent experiments, HaCaT cells were exposed to 60 µM Cr(VI) for 4 hrs. Cr(VI) activated the Akt, NF-κB and MAPK pathways in HaCaT cells. NAC (10 mM) effectively inhibited the activation of phospho-Akt, phospho-p65, phospho-IκBα, phospho-ERK, phospho-p38 and phospho-JNK.

HaCaT cells were treated with NAC alone, Cr(VI) alone or in combination and a whole cell extract was prepared to examine the activation of the Akt, NF-κB, and MAPK pathways. As shown in Fig. 5, treatment of NAC (5 or 10 mM) effectively inhibited the activation of Akt, p65 NF-κB, IκBα (in 5 mM NAC treatment), ERK, p38(in 10 mM NAC treatment), and JNK(in 5 mM NAC treatment). These results suggest that the Cr(VI)-induced p65 NF-κB activation is inhibited by NAC treatment in HaCaT cells. Previous studies showed that Cr(VI) could activate the Akt, NFκB, and MAPK pathways in certain cell types especially in the lungs [3]. In this study, we demonstrated that, in human keratinocytes, Cr(VI) could activate the Akt, NFκB, and MAPK pathways and that pretreatment with NAC could effectively inhibit these activations.

### NAC inhibited the expression of IL-1α and TNF-α mRNA induced by Cr(VI) in HaCaT cells

**Figure 6:**
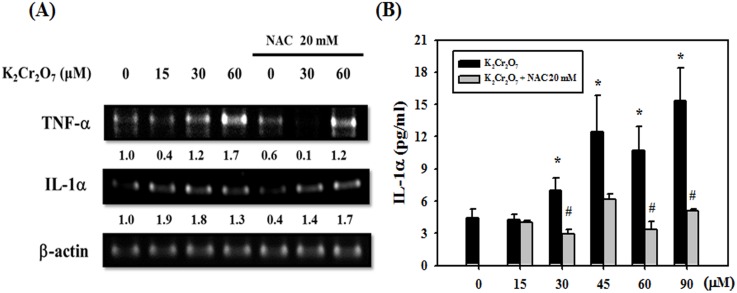
Effects of Cr(VI) and NAC on TNF-α and IL-1α production. (A) Cells were pretreated with NAC (20 mM) for 1 hr and then incubated in the presence of different concentrations of Cr(VI) for 24 hrs. Reverse transcription-PCR analysis shows detectable levels of mRNA in HaCaT cells. NAC suppressed the expression of TNF-α and IL-1α mRNA in HaCaT cells. (B) Conditioned medium samples were collected from HaCaT cells treated with various concentrations of Cr(VI) (15, 30,60, 90 µM). Utilizing ELISA, the levels of IL-1α were measured 24 hrs after treatment. NAC inhibited the release of IL-1α in HaCaT cells. (*Significant versus Control group; p<0.05; #Significant versus Cr(VI) Group; p<0.05).

Utilizing a reverse transcription–polymerase chain reaction (reverse transcription-PCR), HaCaT cells were incubated with Cr(VI) at concentrations of 15, 30 and 60 µM for 4 hrs, reflecting an increase in the expression of TNF-α and IL-1α mRNA in comparison to untreated cells. The increased expression of TNF-α and IL-1α mRNA could be inhibited by pretreatment with NAC (20 mM) (Fig. 6A). Using the ELISA assay, HaCaT cells were treated with various concentrations (15–90 µM) of Cr(VI). The IL-1α levels were measured at 24 hrs after treatment. Fig. 6B showed the results of IL-1α release, which was significantly increased after exposure to Cr(VI). Moreover, pretreatment with NAC could significantly and effectively reduce the release of IL-1α in HaCaT cells.

### NAC administration decreased the activation of the Akt, NF-κB, MAPK pathway and the expression of cytokines in albino guinea pig

**Figure 7:**
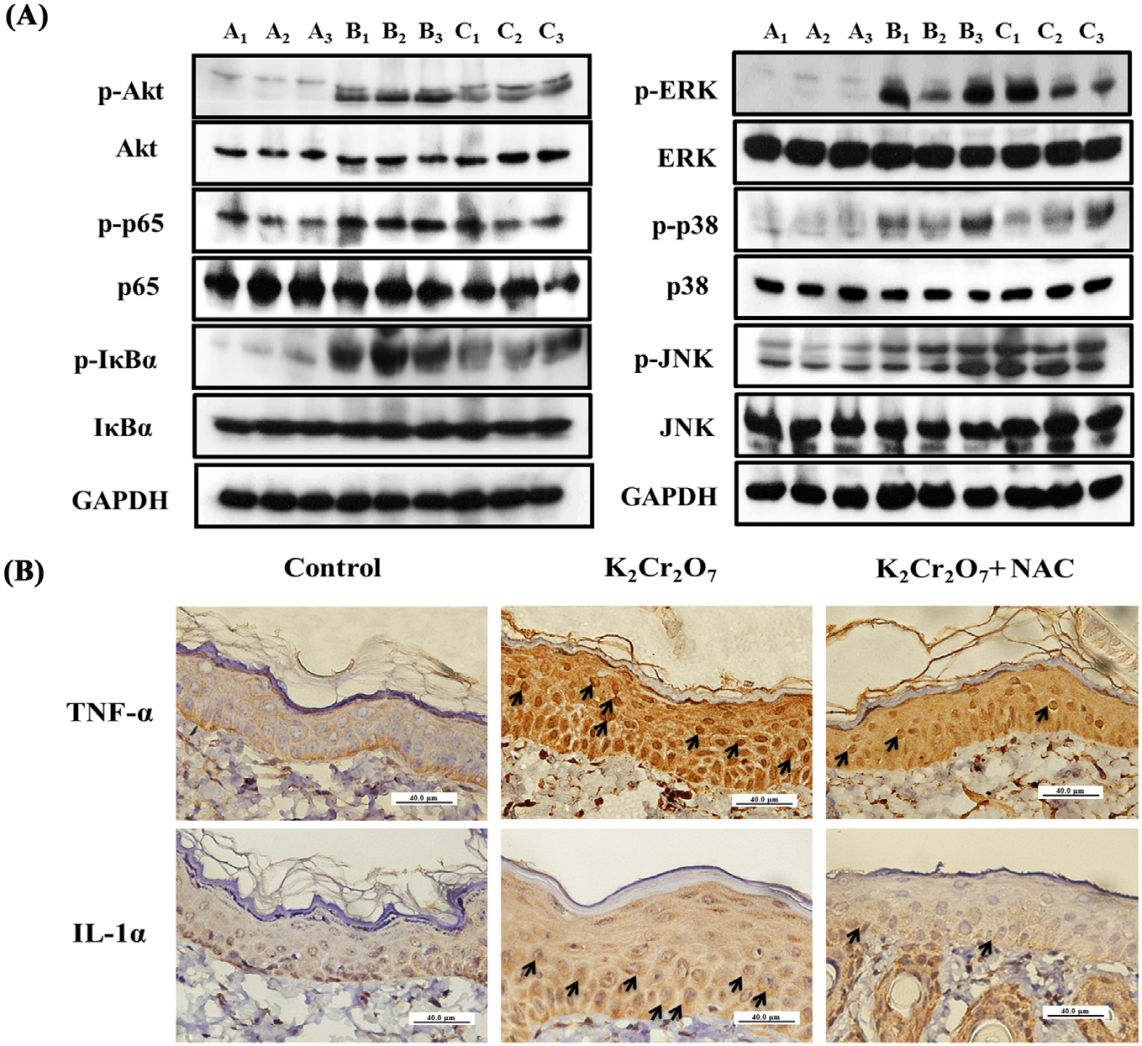
NAC administration decreased the activation of the Akt, NF-κB, MAPK pathway and expression of cytokines in albino guinea pig. Three groups of GP (A, B, and C; n = 3 per group) were used. Group A and Group B were fed only ordinary food, while Group C was fed ordinary food and 1200 mg/kg/day of NAC. Group A were injected with 0.2 ml of saline solution (control group) and Group B and Group C were injected with 2 mg/ml Cr(VI) in saline (Cr(VI) exposed Group). Forty-eight hours after these injections, skin specimens were taken for analysis. (A) Epidermal protein was extracted for western blot analysis and results demonstrated the activation of phospho-Akt, phospho-p65, phospho-IκBα, phospho-ERK, phospho-p38 and phospho-JNK in Group B, but the phosphorylation of these proteins were inhibited by NAC administration. A_1_, A_2_ and A_3_ reflect the three independent specimens from the control group. B_1_, B_2_ and B_3_ reflect the three independent specimens from the Cr(VI) exposed group. C_1_, C_2_ and C_3_ reflect the three independent specimens from the Cr(VI)+NAC exposed group. (B) Skin biopsy from the control group showed no staining of TNF-α and IL-1α in the epidermis (400×). The Cr(VI) exposed group induced the expression of TNF-α (arrow) and IL-1α (arrow) (400×). However, the Cr(VI)+NAC exposed group showed significant reduction in TNF-α and IL-1α in the epidermis (400×). Scale bars: 40 µm.

In our *in* *vivo* study, a dermal injection of Cr(VI) could activate phospho-Akt, phospho-p65, phospho-IκBα, phospho-ERK, phospho-p38, and phospho-JNK in the epidermis of albino GP, but there were no changes in the total Akt, p65, IκBα, ERK, p38, and JNK content. The female albino GPs continued receive NAC for five weeks by gavage or intraperitoneal injection. The results indicated that NAC could significantly suppress the phosphorylation of Akt, NF-κB, and MAPK proteins in the epidermis of albino GP (Fig. 7A). These alterations were consistent with the response in HaCaT cells. Furthermore, the TNF-α and IL-1α expression patterns in skin biopsies were examined using IHC staining. TNF-α and IL-1α were decreased in the skin of albino GP treated with Cr(VI)+NAC compared with Cr(VI) treatment alone (Fig. 7B).

## Discussion

Epidermal keratinocytes provide an essential structural and immunological barrier that forms the first line of defense against potentially pathogenic chemicals and microorganisms. The barrier integrity and innate immune responses in the epidermis are important for the maintenance of skin immune homeostasis and pathogenesis of inflammatory skin diseases [48]. Cell death (apoptosis, necrosis and autophagy) is a highly regulated process and a pivotal mechanism in the maintenance of tissue homeostasis in multicellular organisms. However, uncontrolled cell death can result in numerous pathophysiological conditions, including cancer, neurodegenerative disorders and inflammation [49]–[52]. Among the different cell death types, deregulation of epidermal keratinocyte apoptosis has been demonstrated as a pivotal pathological mechanism in cutaneous inflammatory diseases [19]. Apoptotic cells can carry important and complex information for the regulation of downstream immune response in a context-dependent manner [53]. Unlike apoptosis, autophagy acts as either a survival or death safeguard mechanism in different environmental stresses and cell types. Both apoptosis and autophagy could be observed in keratinocytes treated with Cr(VI) (Figs. 2, 3). Autophagy occurred at an early stage and was observed through the formation of acidic vesicular organelles (the marker for autophagy) and microtubule-associated protein 1 light chain 3-II production. Apoptosis occurred at a later stage and was detected by Annexin V and caspase-3/PARP immunoblotting.

One recent report indicated that autophagy controls inflammation through regulatory interactions with innate immune signaling pathways by removing endogenous inflammasome agonists and effecting on the secretion of immune mediators. Moreover, autophagy contributes to antigen presentation and T cell homeostasis [54]. In addition to apoptosis, we demonstrated for the first time that autophagy could be induced in keratinocytes treated with Cr(VI), in which NAC attenuated the induction of autophagy by interrupting ROS-triggered signaling (Figs. 3, 4). ROS generation induced by Cr(VI) has been well demonstrated in previous studies, including ours [3], [30], [55]. When cells are exposed to excessive ROS, autophagic function may be impaired, resulting in the accumulation of damaged organelles, such as mitochondria, that can induce oxidative stress and inflammation. Finally, this autophagy dysfunction might induce either apoptosis or autophagic cell death to decrease cell viability as observed in our study (Figs. 1–3). Similarly, a recent report indicated that ROS production is a critical reason for Cr(VI)-induced mitochondria-dependent apoptosis. Activation of autophagy could repair mitochondria function to protect hepatocytes by potentially removing damaged mitochondria [56]. Understanding the mechanisms behind ROS-induced autophagy may provide therapeutic implications for skin hypersensitivity. Whether the currently observed autophagy significantly contributes to the process of Cr(VI)-induced hypersensitivity *in* *vivo* remains unclear requires further clarification.

Recently, our group reported that injections of Cr(VI) into GP skin induced skin hypersensitivity through a cytokines-dependent increase in ROS production [3]. These results suggest that Cr(VI) serves as both an antigen and a ROS producer to induce chromium hypersensitivity within this animal model. Thus, antioxidative agents that can reduce the formation of ROS might be a potential approach to prevent chromium hypersensitivity by interfering with the effects of ROS on cytokines (e.g., TNF-a, IL-1) by keratinocytes. In this regard, NAC, one of the most common antioxidants used to investigate the effects of ROS in the pathogenesis of many oxidative stress-related diseases [5], [29], could significantly diminish the skin reaction severity and sensitization rate of chromium hypersensitivity, as well as reducing ROS formation in the skin of coadjuvant chromium-sensitized GP [3]. In certain types of cells, ROS-regulated redox sensitive protein kinases and transcription factors, such as those involved in the Akt, NF-κB and MAPK pathways, might affect the release of cytokines, such as TNF-α and IL-1 [22], [23], [57]. In human keratinocytes, Cr(VI) could increase the formation of ROS, activate the Akt, NF-κB and MAPK pathways, and increase the expression of TNF-α and IL-1α mRNA, and the release of IL-1α. Furthermore, *in* *vivo*, a dermal injection of potassium dichromate could increase the formation of ROS activate the Akt, NF-κB and MAPK pathways and induce the expression of TNF-α and IL-1α in the epidermis of albino GP [30]. The detailed mechanisms of how Cr(VI) impacts the activation of the Akt, NF-κB and MAPK pathways, expression of TNF-α and IL-1α mRNA and release of IL-1α are complicated. However, we believe that the formation of ROS, as generated during the reduction of Cr(VI) to trivalent chromium, is a key factor.

At the molecular level, NAC has been shown to inhibit the activation of C Jun N-terminal kinase, p38 MAP kinase, redox-sensitive activating protein-1 (AP-1) and the NF-κB transcriptional factor, thereby resulting in the suppression of numerous genes regulating the expression of many cytokines, such as TNF-α and IL-1 [21], [22], [29], [58], [59]. We further demonstrated that NAC could attenuate Cr(VI)-induced hypersensitivity through inhibition of apoptosis and autophagy, ROS-related Akt/NF-κB/MAPK signaling and TNF-α/IL-1α cytokine expressions both *in* *vivo* and *in* *vitro*. The expression of TNF-α could increase the release of IL-1α by activating the NF-κB and MAPK pathways [22]. ROS formation can activate the Akt pathway, which is upstream from the NF-κB pathway and can, therefore, induce the activation of NF-κB [21]. The different ROS species appear to mediate different Akt, NF-κB and MAPK downstream signaling pathways [21], [36]. During the processes of Cr(VI) reduction, many ROS, including free radicals, such as the hydroxyl radical, singlet oxygen, superoxide anion are formed [10]. These ROS might regulate redox-sensitive protein kinases and transcription factors to induce toxicity and hypersensitivity [21]. In our current study, we further confirmed that hydrogen peroxide involved in the Cr(VI)-induced hypersensitivity. The antioxidative activity of NAC can be attributed to its reactions with a variety of ROS species, such as ^•^OH, ^•^NO_2_, CO_3_^•−^, thiyl radicals, superoxide, hydrogen-peroxide and peroxynitrite [60]. This could explain why we observe broad-spectrum effects of NAC in attenuating ROS generation, Akt/NF-κB/MAPK signaling and TNF-α/IL-1α expression. Nevertheless, because the activation of each cell signaling pathway is cell type- and stimulus-specific, the detailed mechanisms of the effects of Cr(VI) have not yet been demonstrated in skin cells. Further research is required to clarify the relationship between Cr(VI)-induced ROS formation, cell signaling activation and cytokine release in human keratinocytes.

NAC has been used in clinics for more than 50 years for the treatment of numerous disorders including paracetamol intoxication, acute respiratory distress syndrome, chemotherapy-induced toxicity and heavy metal toxicity [60]. Recently, in a coadjuvant chromium-sensitized GP model, we clearly demonstrated that the use of NAC could significantly reduce the severity of skin reactions and decrease the sensitization rate of chromium hypersensitivity [3]. The mechanisms underlying the therapeutic and clinical applications of NAC are complex and remain unclear. Our current results show that NAC acts through more than one mechanism in preventing Cr(VI)-induced cytotoxicity and hypersensitivity, which can be ROS-dependent. Nonetheless, those findings can only partially explain the diverse biological effects of NAC, and further studies are required for determining its detailed biological functions and elucidating its reactions with components of the cell signaling pathways. While NAC has good efficacy in preventing the progression of chromium hypersensitivity, many factors, including compliance, the cost of NAC and its long-term usage, might compromise its efficiency in clinics. If these limitations could be overcome, NAC has the potential to be used as a chemopreventative agent to prevent the progression of chromium hypersensitivity.
